# Supplementary figures and images for: Activity-dependent modulation of synapse-regulating genes in astrocytes
Source: eLife. 2021 Sep 8;10:e70514. doi: 10.7554/eLife.70514 (PMC8497060; doi:10.7554/eLife.70514)

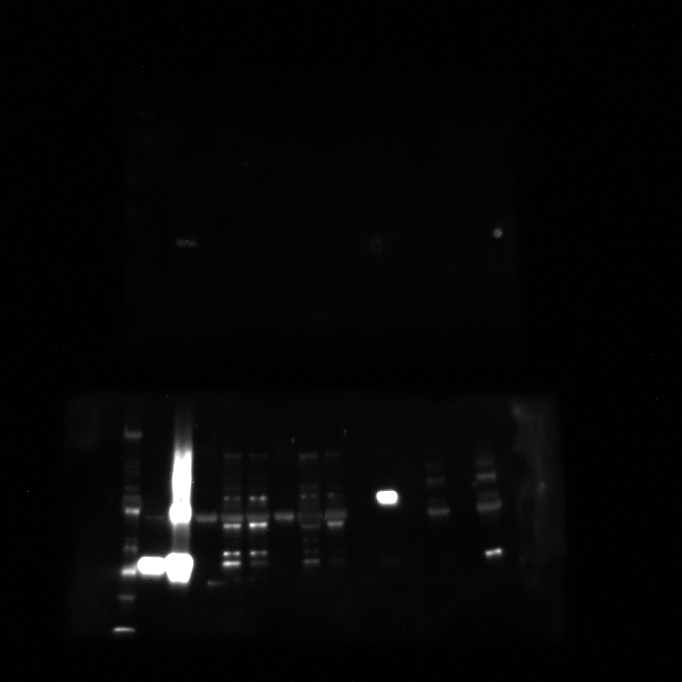

Supplement: Figure 4—source data 1. — (A) Full statistical analysis of mRNA expression differences between WT and KO in VGlut2 cKO model. Averages and analysis calculated for N = 5, i.e. per mouse. (B) Full statistical analysis of mRNA expression differences between WT and KO in VGlut2 cKO model. Averages and analysis calculated for n = ~200–400, that is, total number of astrocytes per group (across five mice). All comparisons are between WT and KO within each layer. [file elife-70514-fig4-data1.zip › Figure 4-figure supplement 1-SourceData1/Figure 4-figure supplement 1-Source Data 1 raw image A.tif]

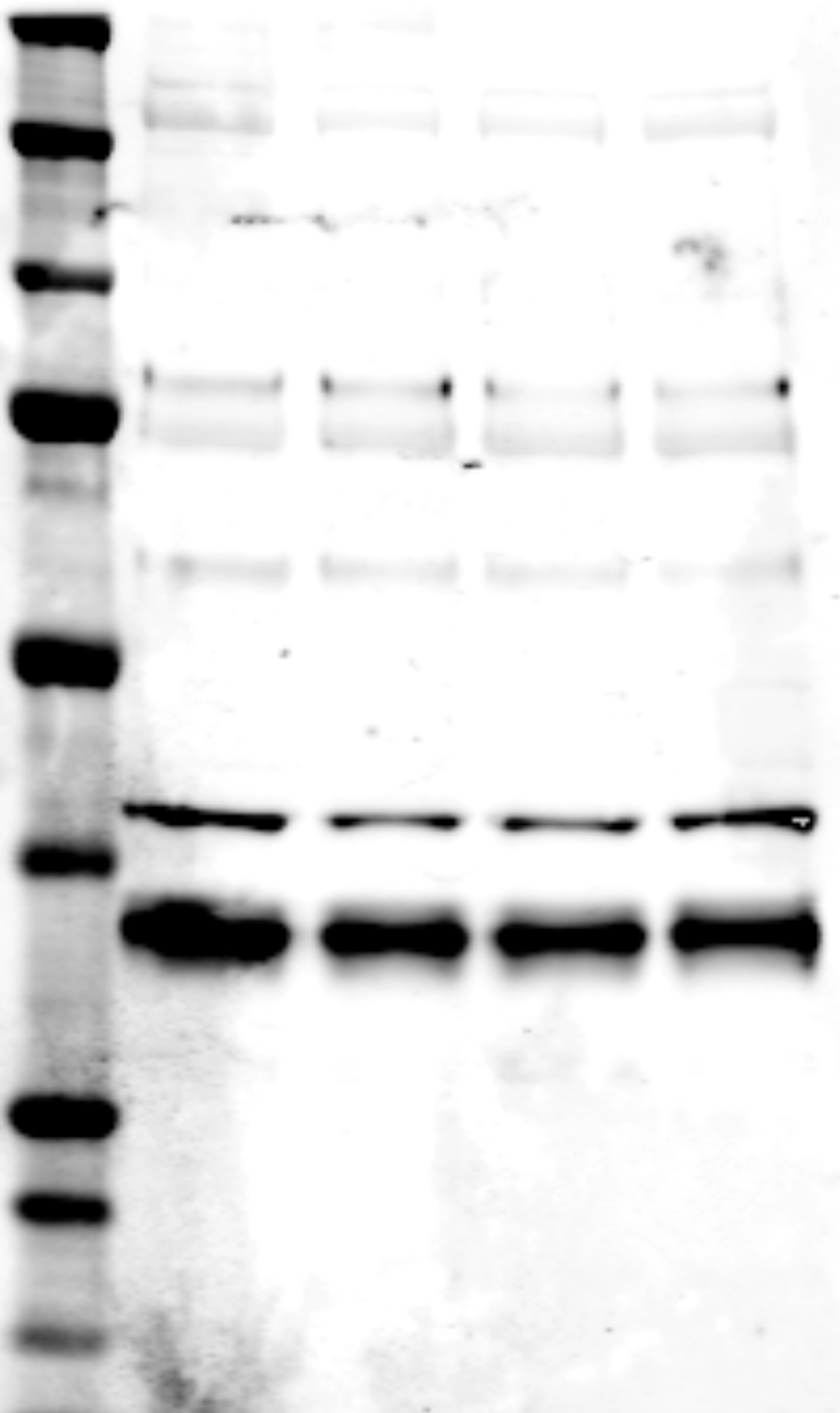

Supplement: Figure 4—source data 1. — (A) Full statistical analysis of mRNA expression differences between WT and KO in VGlut2 cKO model. Averages and analysis calculated for N = 5, i.e. per mouse. (B) Full statistical analysis of mRNA expression differences between WT and KO in VGlut2 cKO model. Averages and analysis calculated for n = ~200–400, that is, total number of astrocytes per group (across five mice). All comparisons are between WT and KO within each layer. [file elife-70514-fig4-data1.zip › Figure 4-figure supplement 1-SourceData1/Figure 4-figure supplement 1-Source Data 1 raw image B left.tif]

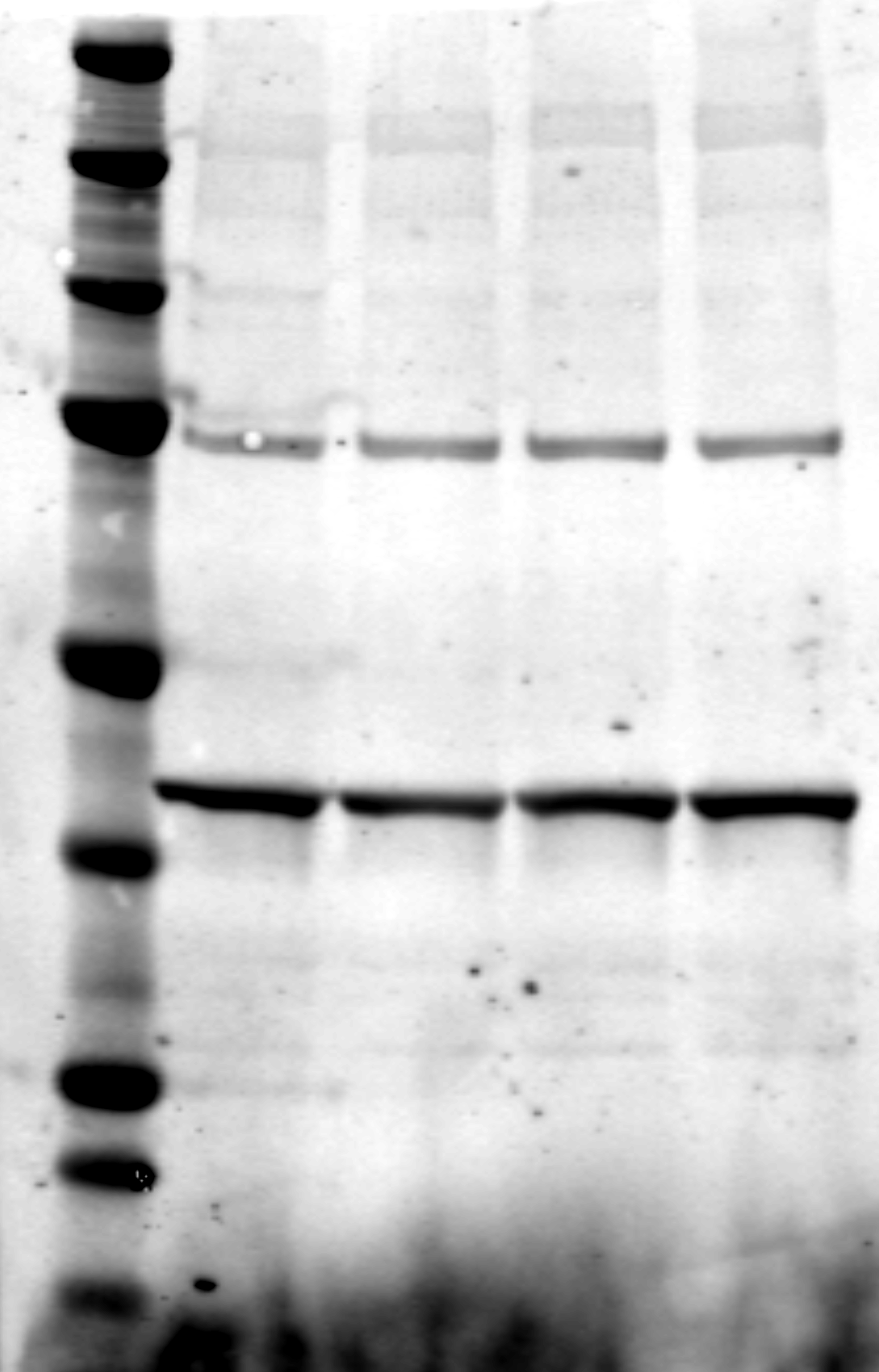

Supplement: Figure 4—source data 1. — (A) Full statistical analysis of mRNA expression differences between WT and KO in VGlut2 cKO model. Averages and analysis calculated for N = 5, i.e. per mouse. (B) Full statistical analysis of mRNA expression differences between WT and KO in VGlut2 cKO model. Averages and analysis calculated for n = ~200–400, that is, total number of astrocytes per group (across five mice). All comparisons are between WT and KO within each layer. [file elife-70514-fig4-data1.zip › Figure 4-figure supplement 1-SourceData1/Figure 4-figure supplement 1-Source Data 1 raw image B right.tif]

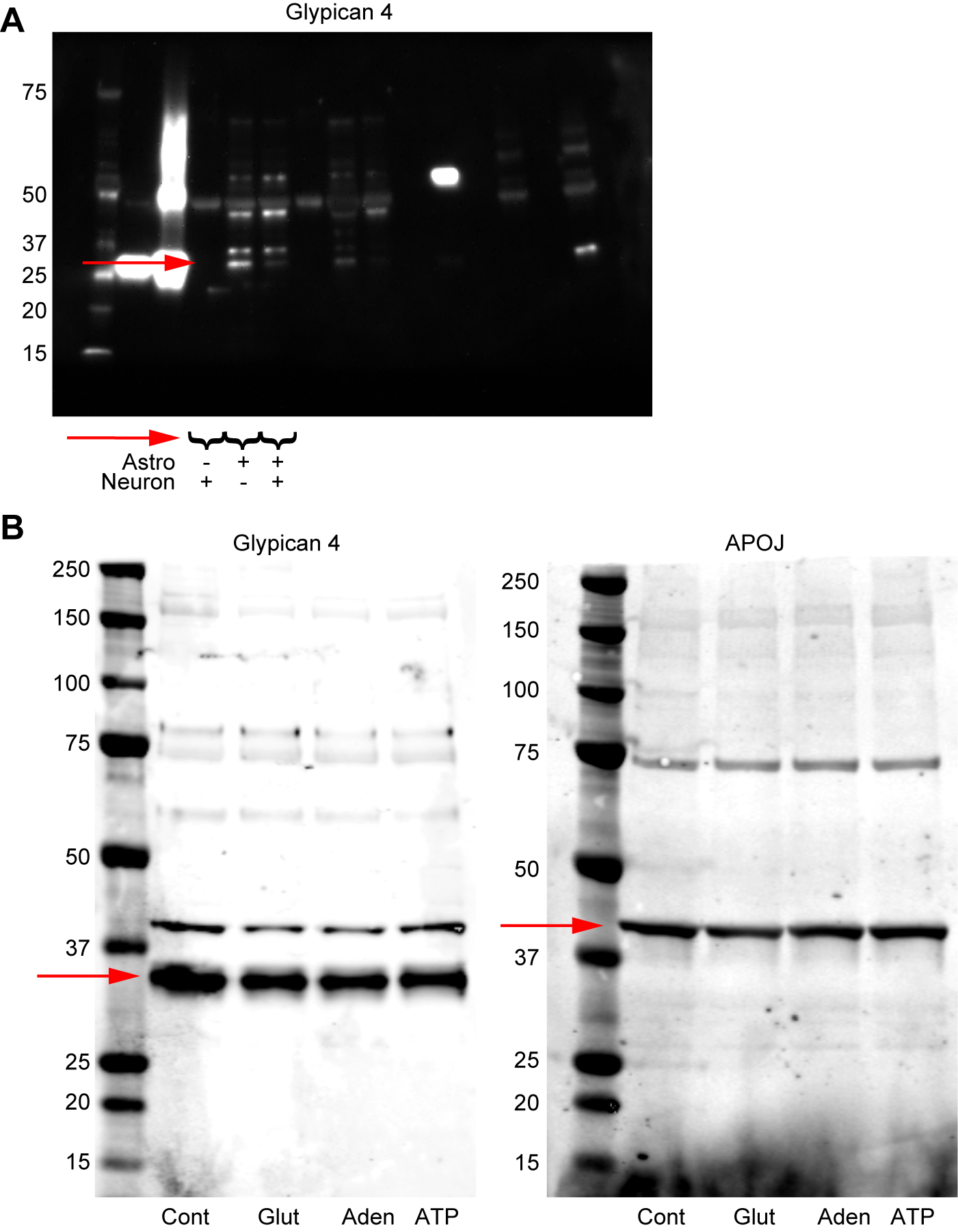

Supplement: Figure 4—source data 1. — (A) Full statistical analysis of mRNA expression differences between WT and KO in VGlut2 cKO model. Averages and analysis calculated for N = 5, i.e. per mouse. (B) Full statistical analysis of mRNA expression differences between WT and KO in VGlut2 cKO model. Averages and analysis calculated for n = ~200–400, that is, total number of astrocytes per group (across five mice). All comparisons are between WT and KO within each layer. [file elife-70514-fig4-data1.zip › Figure 4-figure supplement 1-SourceData1/Figure 4-figure supplement 1-Source Data 1.tif]

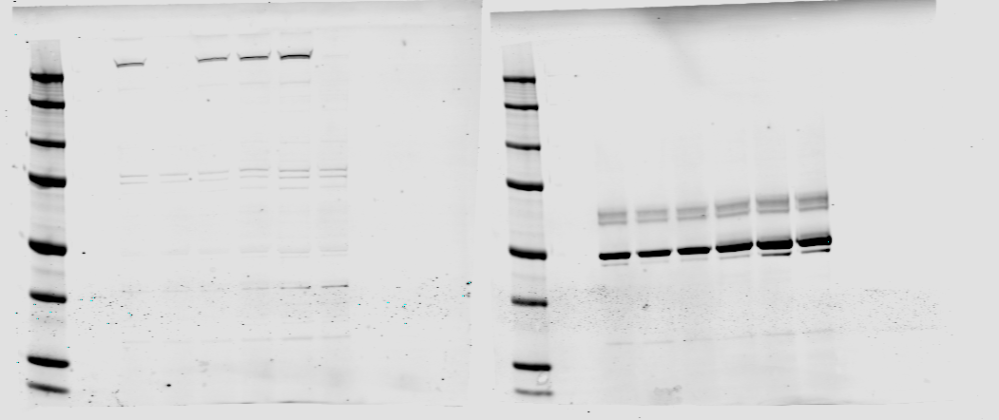

Supplement: Figure 5—source data 2. — Full uncropped western blot representative image showing IP3R2 protein levels are reduced in Ip3r2 KO mice compared to WT. Left panel shows IP3R2, right panel shows β3 tubulin used as loading control. Red arrows indicate IP3R2 signal at ~300 kDa; β3 tubulin signal at ~50 kDa. [file elife-70514-fig5-data2.zip › Figure 5-SourceData2/Figure 5-Source Data 2 raw image.png]

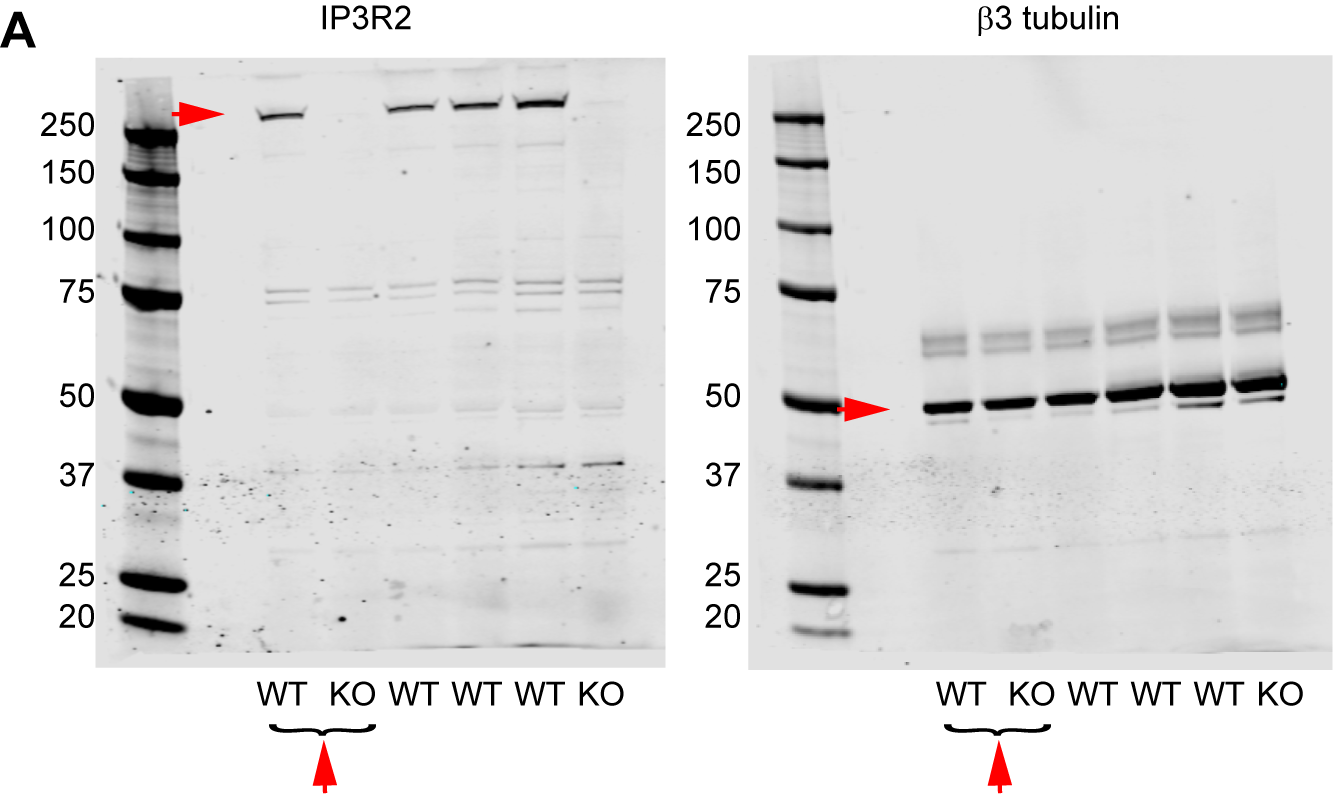

Supplement: Figure 5—source data 2. — Full uncropped western blot representative image showing IP3R2 protein levels are reduced in Ip3r2 KO mice compared to WT. Left panel shows IP3R2, right panel shows β3 tubulin used as loading control. Red arrows indicate IP3R2 signal at ~300 kDa; β3 tubulin signal at ~50 kDa. [file elife-70514-fig5-data2.zip › Figure 5-SourceData2/Figure 5-source data 2.tif]
